# Profiling of Initial Available SARS-CoV-2 Sequences from Iranian Related COVID-19 Patients

**DOI:** 10.22074/cellj.2020.7524

**Published:** 2020-09-08

**Authors:** Najmeh Salehi, Amir Amiri-Yekta, Mehdi Totonchi

**Affiliations:** 1 Department of Genetics, Reproductive Biomedicine Research Center, Royan Institute for Reproductive Biomedicine, ACECR, Tehran, Iran; 2 Department of Bioinformatics, Institute of Biochemistry and Biophysics, University of Tehran, Tehran, Iran; 3 Department of Stem Cells and Developmental Biology, Cell Science Research Center, Royan Institute for Stem Cell Biology and Technology, ACECR, Tehran, Iran

**Keywords:** COVID-19, Nonsynonymous Mutations, SARS-CoV-2, S Protein

## Abstract

The etiologic agent SARS-CoV-2 has caused the outbreak of COVID-19 which is spread widely around the world. It is vital to uncover and investigate the full genome sequence of SARS-CoV-2 throughout the world to track changes in this virus. To this purpose, SARS-CoV-2 full genome sequence profiling of 20 patients in Iran and different countries that already had a travel history to Iran or contacts with Iranian cases were provided from the GISAID database. The bioinformatics analysis showed 44 different nucleotide mutations that caused 26 nonsynonymous mutations in protein sequences with regard to the reference full genome of the SARS-CoV-2 sequence (NC_045512.2). R207C, V378I, M2796I, L3606F, and A6407V in ORF1ab were common mutations in these sequences. Also, some of the detected mutations only were found in Iranian data in comparison with all the available sequences of SARS-CoV-2. The position of S protein mutations showed they were far from the binding site of this protein with angiotensin-converting enzyme-2 (ACE2) as the host cell receptor. These results can be helpful to design specific diagnostic tests, trace the SARS-CoV-2 sequence changes in Iran, and explore therapeutic drugs and vaccines.

## Introduction

Coronaviruses (CoVs) are related to the family of Coronaviridae. They contain a single-stranded RNA of 26 to 32 kilobases. Pathogenic human CoVs usually cause mild respiratory diseases (1). In contrast, two highly pathogenic human CoVs were identified that transmitted from animals to humans. Severe acute respiratory syndrome (SARS) coronavirus (SARSCoV) as the first one was reported in Guangdong, China, in November 2002 that caused more than 8,096 human infections and 774 deaths in 37 countries (2,3). The second one was the Middle East respiratory syndrome (MERS) coronavirus (MERS-CoV), which was first reported in Saudi Arabia in June 2012 that infected 1,728 cases and expired 624 patients in 27 countries (2).

**Fig.1:**
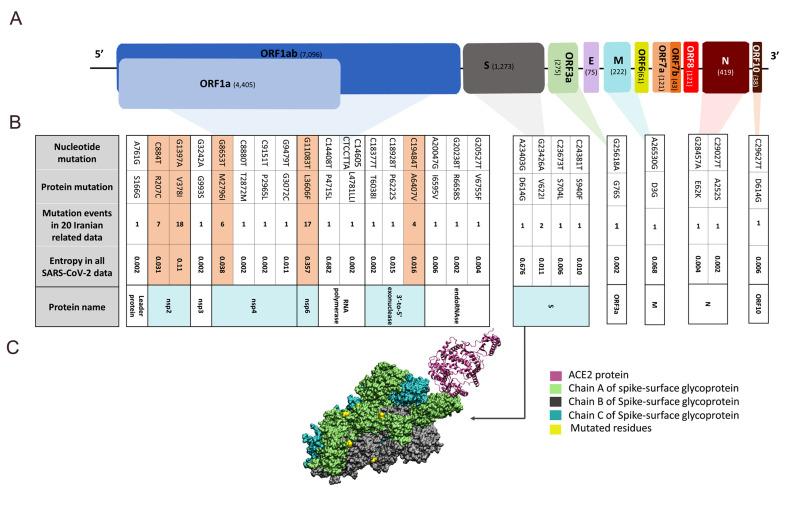
The genomic characterization and the specific mutations in the SARS-CoV-2 sequences of Iranian related COVID-19 patients. **A.** The schematic diagram of the genome organization of SARS-CoV-2 based on the full genome of the SARS-CoV-2 sequence (NC_045512.2). The numbers of the encoded amino acid residues are specified in parenthesis. **B.** The nucleotide and protein mutations, the number of mutation events in data from the 20 Iranian related patients, the entropy values of these mutations in all 3927 SARS-CoV-2 sequences, and the corresponded proteins are depicted. Light orange columns and cyan cells show the common mutations and their corresponded proteins in Iranian related sequences, respectively. **C.** The SARS-CoV-2 and ACE2 complex structure. ACE2, Chain A, B, C of S protein, and mutated residues are depicted in magenta, green, gray, cyan, and yellow, respectively.

In December 2019, a new human coronavirus, SARS-CoV-2, was identified in patients in Wuhan, Hubei Province, China (4,5). This new infectious respiratory disease is called coronavirus disease 19 (COVID-19), which is quickly spread around the world. The COVID-19 outbreak has a total of 2,072,113 infections and 138,475deaths in 210 countries and territories around the world until 15^th^ April 2020. As it can be seen in Figure 1A, the full genome sequence of SARS-CoV-2 has ten open reading frames (ORFs) that contain four structural proteins; the spike-surface glycoprotein (S), the small envelope protein (E), the membrane glycoprotein (M), and the nucleocapsid protein (N), as well as several nonstructural proteins. In all CoVs, the S protein plays a crucial role in binding to the host cell receptors (6,7). A pairwise sequence alignment between the SARS-CoV-2 with SARS-CoV and MERS-CoV showed about 79% and 50% identity, respectively (8). The complete genome profile of SARS-CoV-2 revealed a high overall genome sequence identity to bat-CoV-RaTG13, Pangolin-CoV, bat-SARSr-CoV-ZC45, and bat-SARSr-CoV-ZXC21 by 96.2%, 91.02%, 87·99%, and 87·23%, respectively (8-10) Therefore, SARS-CoV-2 genome is highly similar to RaTG13 genome (9). However, genes such as ORF1b, the S protein, ORF7a, and ORF10 in pangolin-CoV depict higher identity with SARSCoV- 2 than bat-CoV-RaTG13 (10-12).

The genome sequence of SARS-CoV-2 is being generated by a lot of laboratories around the world, and these are freely available at the global initiative on sharing all influenza data (GISAID) database (13). These data can be helpful to design more specific diagnostic tests, trace the ongoing outbreak, and explore therapeutic processes. By 15^th^ April 2020, twenty-three sequences of SARS-CoV-2 with a length of 87 to 595 bases and one full sequence with a length of 29,828 were available at the GISAID database of Iran’s location. Fifteen and eight of these twenty-three sequences were associated with a part of the N gene and the ORF1ab, respectively. Three, four, and one (of eight) sequences of ORF1ab coded a portion of leader protein, RNA polymerase, 3´-to-5´ exonuclease, respectively. All of these twenty-three sequences encoded the related proteins as same as the reference one (NC_045512.2) if we masked the first and last few bases.

On the other hand, nineteen sequences of the full genome sequence of SARS-CoV-2 on the GISAID database from patients in different countries that had a travel history to Iran or contacts with Iranian cases were retrieved from the database. The one full sequence with Iran’s location and these Iranian related sequences were translated to the protein sequence in six frames. The multiple nucleotide and protein sequence alignments of these initial available data were performed by MUSCLE and Clustal Omega programs with default parameters, respectively. The mutation results of nucleotide and protein sequences are available in Table S1 and S2 (See Supplementary Online Information at www.celljournal.org), respectively. As it can be seen in the Table S1, these sequences totally revealed 44 different nucleotide mutations that have made 26 nonsynonymous mutations in protein sequences (Table. S2) regarding the full genome of the SARS-CoV-2 sequence isolate Wuhan-Hu-1 (NC_045512.2). These nucleotide mutations should be noticed in designing diagnostic tests to reduce the false-negative results of no binding of primers and probes in qPCR-based tests. Figure 1B presents nucleotide mutations that lead to nonsynonymous mutations in protein sequences. A six-nucleotide and two-amino-acid insertions were detected in the full genome sequence of SARS-CoV-2 with Iran’s location. The number of mutation events depicts that some of these mutations occurred more than three times among these 20 sequences such as R207C, V378I, M2796I, L3606F and A6407V in ORF1ab which are highlighted in the light orange columns in Figure 1B. Also, the entropy values of these mutations among the 3927 full genome sequences of the SARS-CoV-2 have retrieved from Nextstrain (14) (https://nextstrain.org/) analyses. The entropy values quantify the uncertainty or variability of amino acid mutations for each position in protein sequences. A position on the protein sequence without any mutation in the whole genome of the SARS-CoV-2 sequence has an entropy of zero (15). The entropy values show that some mutations have occurred just once in Iranian sequences were also rare in the 3927 full genome sequences of SARS-CoV-2 with 0.002 entropy value (Fig .1B). Furthermore, the corresponded protein name for each mutation is identified in Figure 1B. Accordingly, nsp2, nsp4, nsp6, 3´-to-5´ exonuclease, endoRNAse, and S protein contain more mutation positions with higher events in data from these 20 Iranian related patients. Among these proteins, the S protein facilitates viral entry into host cells (6, 7).

Similar to SARS-CoV, angiotensin-converting enzyme-2 (ACE2) is used as a cellular entry receptor for SARS-CoV-2 (16,17). The viral replication rates and disease severity depend on the binding affinity between the S protein and the ACE2 receptor (17). The 3D structure of the SARS-CoV-2 receptor-binding domain (RBD) in complex with human ACE2 protein receptor (PDB ID: 6M17) was superimposed on the S protein of SARS-CoV-2 with a single RBD up (PDB ID: 6VSB) by VMD1.9.3 (18) in Figure 1C. As can be seen, the S protein amino-acids variants in our cases are far from the binding site of the S-ACE complex. So, none of these mutations cause any disruption on the binding of S protein with ACE2. On the other hand, for the SARS-CoV-2 vaccine and drug designing the S protein is a perfect target on the surface of this virus (19, 20) which its mutations should be observed.

In this study, the full genome sequences of SARSCoV- 2 from the 20 Iranian related COVID-19 patients were profiled in detail. The results showed some significant mutations such as R207C, V378I, M2796I, L3606F, and A6407V in ORF1ab which occur more than three times among these 20 Iranian related sequences. Also, some rare mutations were found that only happened in these sequences in comparison with all 3927 full genome sequences of SARS-CoV-2. The structural analysis of S protein showed the S protein mutations in Iranian related sequences were away from the binding site of S protein with ACE2. These data can be of great help for performing researches to trace the SARS-CoV-2 sequence changes, designing more specific diagnostic tests to reduce the false-negative results of no binding of primers and probes in qPCRbased tests, as well as exploring specific therapeutic drugs and vaccines in Iran. It is certainly needed to generate more full genome sequences of SARS-CoV-2 from Iranian patients to find more certain changes of this virus in Iran.

## Supplementary PDF



## References

[1] Su S, Wong G, Shi W, Liu J, Lai ACK, Zhou J (2016). Epidemiology, genetic recombination, and pathogenesis of coronaviruses. Trends Microbiol.

[2] De Wit E, Van Doremalen N, Falzarano D, Munster VJ (2016). SARS and MERS: recent insights into emerging coronaviruses. Nat Rev Microbiol.

[3] World Health Organization (2003). Summary of probable SARS cases with onset of illness from 1 November 2002 to 31 July 2003.

[4] Wang C, Horby PW, Hayden FG, Gao GF (2020). A novel coronavirus outbreak of global health concern. Lancet.

[5] Zhu N, Zhang D, Wang W, Li X, Yang B, Song J (2020). A novel coronavirus from patients with pneumonia in China, 2019. N Engl J Med.

[6] Du L, He Y, Zhou Y, Liu S, Zheng BJ, Jiang S (2009). The spike protein of SARS-CoV--A target for vaccine and therapeutic development. Nat Rev Microbiol.

[7] Lu L, Liu Q, Zhu Y, Chan KH, Qin L, Li Y (2014). Structure-based discovery of Middle East respiratory syndrome coronavirus fusion inhibitor. Nat Commun.

[8] Lu R, Zhao X, Li J, Niu P, Yang B, Wu H (2020). Genomic characterisation and epidemiology of 2019 novel coronavirus: implications for virus origins and receptor binding. Lancet.

[9] Zhou P, Yang XL, Wang XG, Hu B, Zhang L, Zhang W (2020). A pneumonia outbreak associated with a new coronavirus of probable bat origin. Nature.

[10] Zhang T, Wu Q, Zhang Z (2020). Probable pangolin origin of SARS-CoV-2 associated with the COVID-19 outbreak. Curr Biol.

[11] Xiao K, Zhai J, Feng Y, Zhou N, Zhang Xu, Zou JJ (2020). Isolation and characterization of 2019-nCoV-like coronavirus from malayan pangolins. bioRxiv.

[12] Zhang J, Jia W, Zhu J, Li B, Xing J, Liao M (2020). Insights into the cross-species evolution of 2019 novel coronavirus. J Infect.

[13] Shu Y, McCauley J (2017). GISAID: Global initiative on sharing all influenza data -from vision to reality. Euro Surveill.

[14] Hadfield J, Megill C, Bell SM, Huddleston J, Potter B, Callender C (2018). NextStrain: Real-time tracking of pathogen evolution. Bioinformatics.

[15] Sherwin WB (2010). Entropy and information approaches to genetic diversity and its expression: Genomic geography. Entropy.

[16] Li W, Moore MJ, Vasllieva N, Sui J, Wong SK, Berne MA (2003). Angiotensin-converting enzyme 2 is a functional receptor for the SARS coronavirus. Nature.

[17] Hoffmann M, Kleine-Weber H, Schroeder S, Krüger N, Herrler T, Erichsen S (2020). SARS-CoV-2 cell entry depends on ACE2 and TMPRSS2 and is blocked by a clinically proven protease inhibitor. Cell.

[18] Humphrey W, Dalke A, Schulten K (1996). VMD: Visual molecular dynamics. J Mol Graph.

[19] Amanat F, Krammer F (2020). SARS-CoV-2 vaccines: status report. Immunity.

[20] Ahmed SF, Quadeer AA, McKay MR (2020). Preliminary identification of potential vaccine targets for the COVID-19 coronavirus (SARS-CoV-2) based on SARS-CoV immunological studies. Viruses.

