# Supplementary material for: Profiling of Initial Available SARS-CoV-2 Sequences from Iranian Related COVID-19 Patients
Source: Cell J. 2020 Sep 8;22(Suppl 1):148–50. doi: 10.22074/cellj.2020.7524 (PMC7481902; doi:10.22074/cellj.2020.7524)
Supplement: Supplementary file 1 [file Cell-J-22-Suppl1-148-s01.pdf]

www.celljournal.org

Supplementary Information for

Profiling of Initial Available SARS-CoV-2 Sequences from Iranian Related COVID-19 Patients

Najmeh Salehi, Ph.D.[1, 2], Amir Amiri-Yekta, Ph.D.[1], Mehdi Totonchi, Ph.D.[1, 3*]

1. Department of Genetics, Reproductive Biomedicine Research Center, Royan Institute for Reproductive Biomedicine, ACECR, Tehran, Iran
2. Department of Bioinformatics, Institute of Biochemistry and Biophysics, University of Tehran, Tehran, Iran
3. Department of Stem Cells and Developmental Biology, Cell Science Research Center, Royan Institute for Stem Cell Biology and Technology, ACECR, Tehran, Iran

**Corresponding Address: P.O.Box: 16635-148, Department of Genetics, Reproductive Biomedicine Research Center, Royan Institute for Reproductive Biomedicine, ACECR, Tehran, Iran*
Email: m.totonchi@royaninstitute.org

Table S1: The results of multiple nucleotide sequence alignments of the Iranian related full genome sequences of SARS-CoV-2

**ID/Name**	**ORF1ab**																													**Surface glycoprotein**					**ORF3a**		**Membrane glycoprotein**	**Nucleocapsid phosphoprotein**				**Non-Coding**	**ORF10**	**Non-Coding**
**Mutations**	**C241T**	**C313T**	**A761G**	**C835T**	**C884T**	**C1348T**	**G1397A**	**C1825T**	**C2113T**	**C3037T**	**G3242A**	**G4255A**	**C7735T**	**G8653T**	**C8880T**	**C9159T**	**G9479T**	**A9514G**	**T9931C**	**C11074T**	**G11083T**	**C14408**	**C14605 CTCCTTA**	**C18377T**	**C18928T**	**C19484T**	**A20047G**	**G20238T**	**G20527T**	**A23403G**	**G23426A**	**C23673T**	**C24381T**	**C24904T**	**G25618A**	**A25980C**	**A26530G**	**G28457A**	**T28688C**	**C29027T**	**G29374A**	**G29543T**	**C29627T**	**G29742T**
hCoV-19/Canada/BC_37_0-2/2020|EPI_ISL_412965	-	-	-	-	-	-	✓	-	-	-	-	-	-	-	-	-	-	✓	-	-	-	-	-	-	-	-	-	-	-	-	-	-	-	-	-	-	-	-	✓	-	-	-	-	✓
hCoV-19/Australia/NSW05/2020|EPI_ISL_412975	-	-	-	-	-	-	✓	-	-	-	-	✓	-	-	-	-	-	-	-	-	✓	-	-	-	-	-	✓	-	-	-	-	-	-	-	-	-	-	-	✓	-	-	-	-	✓
hCoV-19/Australia/NSW06/2020|EPI_ISL_413213	-	-	-	-	✓	-	✓	-	-	-	-	-	-	✓	-	-	-	-	-	-	✓	-	-	-	-	-	-	-	-	-	-	-	-	✓	-	-	-	-	✓	-	-	-	-	✓
hCoV-19/New Zealand/01/2020|EPI_ISL_413490	-	-	-	-		-	✓	-	-	-	-	-	-	-	-	-	-	-	-	-	✓	-	-	-	-	-	-	-	-	-	-	-	-	-	✓	-	-	-	✓	-	-	-	-	✓
hCoV-19/Australia/NSW11/2020|EPI_ISL_413597	-	-	-	-	✓	-	✓	-	-	-	-	-	-	✓	-	-	-	-	-	-	✓	-	-	-	-	-	-	-	-	-	-	-	-	-	-	-	-	-	✓	-	-	-	-	-
hCoV-19/Australia/NSW12/2020|EPI_ISL_413598	-	-	-	-	✓	-	✓	-	-	-	-	-	-	✓	-	-	-	-	-	-	✓	-	-	-	-	-	-	-	-	-	-	-	✓	-	-	-	-	✓	✓	-	-	-	✓	-
hCoV-19/Australia/NSW13/2020|EPI_ISL_413599	-	-	-	-	-	-	✓	-	✓	-	-	-	-	-	-	-	-	-	-	-	✓	-	-	-	✓	-	-	-	-	-	-	-	-	-	-	-	-	-	✓	-	✓	-	-	-
hCoV-19/USA/NY1-PV08001/2020|EPI_ISL_414476	-	-	-	-	-	-	✓	-	-	-	✓	-	-	-	-	-	-	✓		-	✓	-	-	-	-	-	-	-	-	-	-	-	-	-	-	-	-	-	✓	✓	-	-	-	✓
hCoV-19/Germany/BavPat2/2020|EPI_ISL_414520	-	-	-	-	-	-	✓	-	-	-	-	-	-	-	✓	-	✓	✓	✓	-	✓	-	-	-	-	-	-	-	-	-	-	-	-	-	-	-	-	-	✓	-	-	-	-	✓
hCoV-19/Finland/FIN-266/2020|EPI_ISL_414646	✓	-	-	-	-	-	-	-	-	✓	-	-	-	-	-	-	-		-	-	-	✓	-	-	-	-	-	-	-	✓	-	-	-	-	-	-	✓	-	-	-	-	-	-	-
hCoV-19/Canada/BC_69243/2020|EPI_ISL_415577	-	-	-	-	-	-	✓	-	-	-	-	-	-	-	-	-	-	✓	-	-	✓	-	-	-	-	✓	-	-	✓	-	-	-	-	-	-	-	-	-	✓	-	-	-	-	✓
hCoV-19/Canada/BC_13297/2020|EPI_ISL_415578	-	✓	-	-	-	-	✓	-	-	-	-	-	-	-	-	-	-	-	-	-	✓	-	-	-	-	✓	-	✓	-	-	-	-	-	-	-	-	-	-	✓	-	-	-	-	✓
hCoV-19/Canada/BC_25211/2020|EPI_ISL_415579	-	-	-	-	✓	-	✓	✓	-	-	-	-	✓	✓	-	-	-	-	-	✓	✓	-	-	-	-		-	-	-	-	-	-	-	-	-	✓	-	-	✓	-	-	-	-	✓
hCoV-19/Canada/BC_17397/2020|EPI_ISL_415580	-	-	✓	-	-	-	✓	-	-	-	-	-	-	-	-	-	-	-	-	-	✓	-	-	-	-	✓	-	-	-	-	-	✓	-	-	-	-	-	-	✓	-	-	-	-	✓
hCoV-19/Canada/BC_02421/2020|EPI_ISL_415581	-	-	-	-	-	-	✓	-	-	-	-	-	-	-	-	-	-	-	-	-	✓	-	-	-	-	✓	-	-	-	-	-	-	-	-	-	-	-	-	✓	-	-	-	-	✓
hCoV-19/Canada/BC_40860/2020|EPI_ISL_415583	-	-	-	-	-	-	-	-	-	-	-	-	-	-	-	-	-	-	-	-	-	-	-	-	-	-	-	-	-	-	-	-	-	-	-	-	-	-	-	-	-	-	-	✓
hCoV-19/Canada/BC_65034/2020|EPI_ISL_415585	-	-	-	✓	✓	-	✓	-	-	-	-	-	-	✓	-	-	-	-	-	-	✓	-	-	-	-	-	-	-	-	-	✓	-	-	-	-	-	-	-	✓	-	-	✓	-	✓
hCoV-19/Canada/BC_66353/2020|EPI_ISL_415587	-	-	-	✓	✓	-	✓	-	-	-	-	-	-	✓	-	-	-	-	-	-	✓	-	-	-	-	-	-	-	-	-	✓	-	-	-	-	-	-	-	✓	-	-	-	-	✓
hCoV-19/Pakistan/Gilgit1/2020|EPI_ISL_417444	✓	-	-	-	✓	✓	✓	-	-	-	-	-	-	-	-	✓	-	-	-	-	✓	-	-	-	-	-	-	-	-	-	-	-	-	-	-	-	-	-	-	-	-	-	-	-
hCoV-19/Iran/HGRC-01-IPI-8206/2020/EPI_ISL_424349	-	-	-	-	-	-	✓	-	-	-	-	-	-	-	-	-	-	-	-	-	✓	-	✓	✓	-	-	-	-	-	-	-	-	-	-	-	-	-	-	-	-	✓	-	-	✓

Table S2: The results of multiple protein sequence alignments of Iranian related data

ID/Name	ORF1ab																Surface glycoprotein				ORF3a	Membrane glycoprotein	Nucleocapsid phosphoprotein		ORF10
**Mutations**	**S166G**	**R207C**	**V378I**	**G993S**	**M2796I**	**T2872M**	**P2965L**	**G3072C**	**L3606F**	**P4715L**	**L4781LLI**	**T6038I**	**P6222S**	**A6407V**	**I6595V**	**R6658S**	**V6755F**	**D614G**	**V622I**	**S704L**	**S940F**	**G76S**	**D3G**	**E62K**	**A252S**	**R24C**
hCoV-19/Canada/BC_37_0-2/2020|EPI_ISL_412965	-	-	✓	-	-	-	-	-	-	-	-	-	-	-	-	-	-	-	-	-	-	-	-	-	-	-
hCoV-19/Australia/NSW05/2020|EPI_ISL_412975	-	-	✓	-	-	-	-	-	✓	-	-	-	-	-	✓	-	-	-	-	-	-	-	-	-	-	-
hCoV-19/Australia/NSW06/2020|EPI_ISL_413213	-	✓	✓	-	✓	-	-	-	✓	-	-	-	-	-	-	-	-	-	-	-	-	-	-	-	-	-
hCoV-19/New Zealand/01/2020|EPI_ISL_413490	-	-	✓	-	-	-	-	-	✓	-	-	-	-	-	-	-	-	-	-	-	-	✓	-	-	-	-
hCoV-19/Australia/NSW11/2020|EPI_ISL_413597	-	✓	✓	-	✓	-	-	-	✓	-	-	-	-	-	-	-	-	-	-	-	-	-	-	-	-	-
hCoV-19/Australia/NSW12/2020|EPI_ISL_413598	-	✓	✓	-	✓	-	-	-	✓	-	-	-	-	-	-	-	-	-	-	-	✓	-	-	✓	-	✓
hCoV-19/Australia/NSW13/2020|EPI_ISL_413599	-	-	✓	-	-	-	-	-	✓	-	-	-	✓	-	-	-	-	-	-	-	-	-	-	-	-	-
hCoV-19/USA/NY1-PV08001/2020|EPI_ISL_414476	-	-	✓	✓	-	-	-	-	✓	-	-	-	-	-	-	-	-	-	-	-	-	-	-	-	✓	-
hCoV-19/Germany/BavPat2/2020|EPI_ISL_414520	-	-	✓	-	-	✓	-	✓	✓	-	-	-	-	-	-	-	-	-	-	-	-	-	-	-	-	-
hCoV-19/Finland/FIN-266/2020|EPI_ISL_414646	-	-	-	-	-	-	-	-	-	✓	-	-	-	-	-	-	-	✓	-	-	-	-	✓	-	-	-
hCoV-19/Canada/BC_69243/2020|EPI_ISL_415577	-	-	✓	-	-	-	-	-	✓	-	-	-	-	✓	-	-	✓	-	-	-	-	-	-	-	-	-
hCoV-19/Canada/BC_13297/2020|EPI_ISL_415578	-	-	✓	-	-	-	-	-	✓	-	-	-	-	✓	-	✓	-	-	-	-	-	-	-	-	-	-
hCoV-19/Canada/BC_25211/2020|EPI_ISL_415579	-	✓	✓	-	✓	-	-	-	✓	-	-	-	-	-	-	-	-	-	-	-	-	-	-	-	-	-
hCoV-19/Canada/BC_17397/2020|EPI_ISL_415580	✓	-	✓	-	-	-	-	-	✓	-	-	-	-	✓	-	-	-	-	-	✓	-	-	-	-	-	-
hCoV-19/Canada/BC_02421/2020|EPI_ISL_415581	-	-	✓	-	-	-	-	-	✓	-	-	-	-	✓	-	-	-	-	-	-	-	-	-	-	-	-
hCoV-19/Canada/BC_40860/2020|EPI_ISL_415583	-	-	-	-	-	-	-	-	-	-	-	-	-	-	-	-	-	-	-	-	-	-	-	-	-	-
hCoV-19/Canada/BC_65034/2020|EPI_ISL_415585	-	✓	✓	-	✓	-	-	-	✓	-	-	-	-	-	-	-	-	-	✓	-	-	-	-	-	-	-
hCoV-19/Canada/BC_66353/2020|EPI_ISL_415587	-	✓	✓	-	✓	-	-	-	✓	-	-	-	-	-	-	-	-	-	✓	-	-	-	-	-	-	-
hCoV-19/Pakistan/Gilgit1/2020|EPI_ISL_417444	-	✓	✓	-	-	-	✓	-	✓	-	-	-	-	-	-	-	-	-	-	-	-	-	-	-	-	-
hCoV-19/Iran/HGRC-01-IPI-8206/2020/EPI_ISL_424349	-	-	✓	-	-	-	-	-	✓	-	✓	✓	-	-	-	-	-	-	-	-	-	-	-	-	-	-
